# The *Helicobacter pylori* TlpD cytoplasmic chemoreceptor requires an intact C-terminus for polar localization and function

**DOI:** 10.1128/jb.00394-25

**Published:** 2026-02-09

**Authors:** Raymondo Lopez-Magaña, Karen M. Ottemann

**Affiliations:** 1 Department of Microbiology and Environmental Toxicology, University of California Santa Cruz 542823, Santa Cruz, California, USA; National Institutes of Health, Bethesda, Maryland, USA

**Keywords:** localization, chemotaxis, chemoreceptor

## Abstract

Bacteria localize proteins to distinct subcellular locations, including chemoreceptors, which frequently localize to the bacterial pole. Although some polarity-promoting mechanisms have been described, many chemoreceptors lack clear routes to becoming polar. TlpD of the bacterial pathogen *Helicobacter pylori* is one such protein. This cytoplasmic chemoreceptor localizes to the pole in a manner that is independent of the other chemoreceptors. In this work, we evaluated the role of TlpD domains in its function. Truncated proteins were created that lacked different amounts of the N- or C-termini and expressed in *H. pylori* in place of native *tlpD* or as the sole chemoreceptor. These TlpD variants were examined for their presence and abundance, protein localization, association with chemotaxis signaling proteins, and effect on motility. TlpD that lacked any portion of the N-terminal 104 amino acids produced low to no amounts of detectable protein. In contrast, TlpD was detectable with loss of the C-terminal 45 amino acids. TlpD lacking the last 45 amino acids (TlpD∆C4) preserved the ability to interact with CheW and CheV proteins based on bacterial two-hybrid analysis, but was unable to localize to the pole either on their own or in the presence of other chemoreceptors. TlpD∆C4 was found to be diffuse in the cytoplasm and interacted with CheV1, CheV2, and CheV3 at this location but not with CheW. TlpD∆C4 did not confer chemotactic abilities in soft agar chemotaxis assays. These findings suggest the C-terminal end of TlpD plays a previously unappreciated role in promoting TlpD polar localization and function.

**IMPORTANCE:**

Bacteria place their proteins in specific locations that are required for the proteins to function, including the bacterial pole. How the bacterial cell identifies which proteins go to the pole is not fully understood. In this work, we dissect parts of a protein called TlpD that naturally goes to the pole. We find that mutants lacking one end of TlpD lose their polar placement, but retain other abilities. TlpD allows directed motility known as chemotaxis. This ability is critical for infection in *Helicobacter pylori* and numerous other pathogens. When TlpD loses its polar placement, the protein no longer functions for chemotaxis, laying the foundation for future studies that can dissect how this segment promotes function and eventually translate into therapies for *H. pylori* infection.

## INTRODUCTION

*Helicobacter pylori* is a human pathogen that infects approximately one-half of the world’s population (1). This bacterium infects the stomach and causes chronic stomach inflammation and gastric cancer (1). To reach the stomach, *H. pylori* must navigate a dynamic environment. En route, *H. pylori* encounters various signals, including mammalian-produced ones such as acid from stomach parietal cells and reactive oxygen from epithelial and innate immune cells (2–5). One strategy *H. pylori* uses to circumvent such harmful compounds and find beneficial ones is directed motility, also known as chemotaxis (6). The chemotaxis signal transduction system, extensively studied in *Escherichia coli*, allows bacteria to sense concentration gradients of particular chemicals and initiate a motility response (7–10). This chemotaxis system is required for *H. pylori* to establish infection (2, 11–14). The ability to sense and mount an appropriate response towards these signals allows effective navigation and infection.

The *H. pylori* chemotaxis system, like the *E. coli* one, operates as a multiprotein complex. Like that of well-studied bacteria, this complex forms via protein-protein interactions, including between sensors known as chemoreceptors that form interactions with coupling or scaffold proteins and the CheA histidine kinase, to control kinase activity (15–17). In contrast to the one-coupling protein system of *E. coli* that uses the canonical coupling protein CheW, the chemotaxis system of *H. pylori* contains three additional coupling proteins called CheV1, CheV2, and CheV3 (15, 18). These CheV paralogs are proposed to function in several ways, including to generally couple receptor sensing to kinase control, as phosphate sinks, or possibly as unique coupling proteins to accommodate chemoreceptors that cannot couple to CheW (15, 19, 20). In the *H. pylori* system, both CheW and CheV1 are critical to build chemoreceptor arrays (16, 17), and each CheV plays specific roles in motility (18, 21). However, the exact roles of CheV2 and CheV3 in *H. pylori* chemotaxis have yet to be determined (15, 16).

The *H. pylori* chemotaxis system uses four chemoreceptors called TlpA, TlpB, TlpC, and TlpD, proteins that are defined by the methyl-accepting (MA) domain, which interacts with the coupling proteins. TlpA, TlpB, and TlpC are integral membrane proteins, whereas TlpD is soluble, not membrane-bound, and cytoplasmic (6, 22). This cytoplasmic chemoreceptor contains a C-terminal chemoreceptor zinc binding (CZB) sensing domain (17, 23). Despite being cytoplasmic, TlpD localizes to the pole and retains this capability even in the absence of any membrane-bound chemoreceptors (17). However, the mechanism by which TlpD locates to the cell pole remains unclear.

TlpD recruits other chemotaxis proteins to the cell pole. This chemoreceptor is sufficient to recruit CheA, CheW, CheV1, and CheV3 proteins to the *H. pylori* cell pole (17). TlpD likely interacts with the CheW and CheV coupling proteins via its (MA) domain, as has been shown in other chemoreceptors. TlpD has been shown to interact directly with coupling proteins CheW and CheV1 (16). This interaction with coupling proteins facilitates an indirect interaction with the CheA kinase to form a functional signaling unit. Indeed, a combination of reconstituted TlpD, CheA, and CheW or CheV1 proteins is sufficient to drive CheA phosphorylation and suggests either coupling protein can work with TlpD to create a functional signaling unit *in vitro* (16, 24). Moreover, *H. pylori* with TlpD as its only chemoreceptor retains spreading motility on soft agar, indicative of chemotaxis function, and thus supports a model by which TlpD alone is able to form an autonomous and functional signaling unit (17).

TlpD plays a key role in *H. pylori* infection. Numerous studies have shown that TlpD is required for full stomach colonization in both mice and gerbils (2, 11, 25). *tlpD* mutants colonize less well than wild type (WT) or any isogenic other chemoreceptor mutant (2, 11, 25). Additionally, TlpD mounts a chemotactic response to reactive oxygen species, including hydrogen peroxide, superoxide, and hypochlorous acid (17, 24). As all these signals are found during infection, it makes sense that TlpD would be an important chemoreceptor. Indeed, TlpD was found to be expressed in all tested *H. pylori* strains, suggesting it is retained to a higher degree than other chemoreceptors (17). These findings highlight the importance of TlpD in the chemotaxis system of *H. pylori* for infection.

In this study, we evaluated the roles of parts of TlpD for which there is no known function. We created TlpD truncations lacking portions of either the N or C terminus and analyzed how loss of these sequences affected function *in situ*, producing the proteins in the native *H. pylori*. Most truncations resulted in loss of detectable TlpD in *H. pylori*, but one mutant construct led to detectable protein in *H. pylori*: a C-terminus deletion of the last 45 amino acids, 388–433. This region is C-terminal to the CZB domain, and based on bioinformatic analysis, had no detectable features or domains. This truncated protein, called TlpD∆C4, maintained its ability to interact with CheW and CheV coupling proteins, based on immunolocalization and bacterial two-hybrid assays, but lost its ability to localize to the pole and function.

## RESULTS

### Identification of TlpD domain boundaries to allow selective omission of unannotated regions

**Fig 1:**
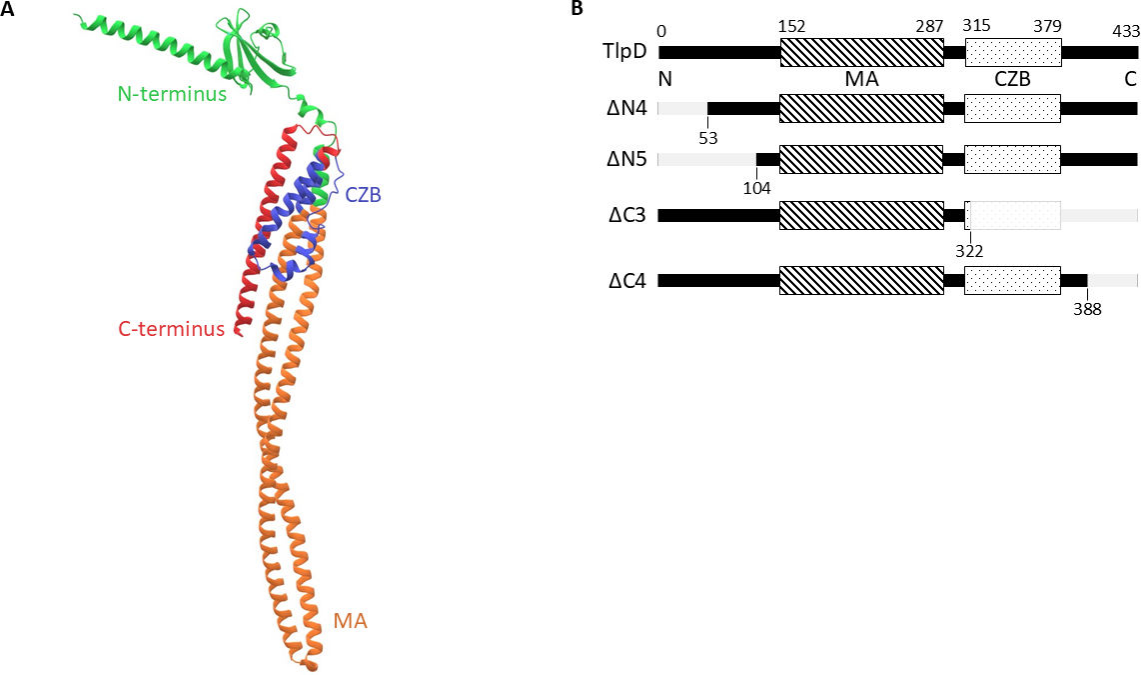
*H. pylori* TlpD predicted structure and domains. (**A**) AlphaFold predicted structure of *H. pylori* TlpD. TlpD is 433 amino acids in length with two known domains, the MA domain (orange) and the CZB domain (blue). The region with no known domains at the N-terminus is shown in green, and at the C-terminus in red. (**B**) Diagram of modified versions of *H. pylori* TlpD with domains and truncation sites indicated (gray). TlpD represents the full-length version of the protein at 433 amino acids, ΔN4 is truncated at residue 53, ΔN5 is truncated at residue 104, ΔC3 is truncated at residue 322, and ΔC4 is truncated at residue 388. Each of these constructs contains a C-terminal 3xFLAG tag.

Previous work had identified domains of TlpD (23, 24), but to check for new annotations and define precise boundaries, we carried out up-to-date bioinformatic analyses. AlphaFold2 (26) predicted the known MA and CZB domains (23uyl) and provided a predicted structure of the 433 amino acid TlpD protein (Fig. 1A). AlphaFold2 defined the MA domain (InterPro accession number: IPR004089) (27) as amino acids 152–287 and the CZB (IPR025991) (27) domain from 315 to 379 (Fig. 1B). Following the C-terminal CZB domain was a 54 amino acid stretch with no annotated domains (Fig. 1A and B). The N-terminus comprises the first 140 amino acids and is predicted to orient perpendicular to the rest of the protein (Fig. 1A and B). Like the C-terminus, the N-terminal stretch had no annotated motifs (Fig. 1A). Further analysis using MotifFinder (28) and NCBI Conserved Domain Database (29) searches with default parameters failed to identify any known domain or motifs in the N- or C-terminal regions (File S1). Overall, these analyses suggest that TlpD has MA and CZB domains in the middle portion, while the N- and C-termini are predicted to be folded into secondary structures, but no known domains were identified using the methods applied in this study.

Because it is unclear what the functions of the TlpD N- or C-terminus are, we took advantage of the two-domain architecture of TlpD to create truncated versions that removed select regions outside of the MA or CZB domains (Fig. 1B). An initial round of TlpD truncation constructs resulted in proteins that were not produced in *H. pylori* at all, possibly because they did not carefully avoid predicted secondary structure endpoints (Fig. S1). In the second round, predicted secondary structure endpoints were more carefully avoided using the information collected above, and a 3× FLAG tag was added to improve detection (File S1 and S2). These constructs were designed to be expressed in *H. pylori* from single copy on the chromosome under the control of the *tlpD* promoter, by integration into the non-essential *rdxA* locus of *H. pylori* (Fig. S2A; Tables 1 and 2).

**TABLE 1:** Plasmids used in this study

Plasmid	KO reference no.	Genotype/description	Reference/source
DH10B pTwist *tlpD 3xflag*	KO1926	*rdxA::tlpD 3xflag erm*	This study
DH10B pTwist *tlpDΔN4*	KO1927	*rdxA::tlpDΔN4 3xflag erm*	This study
DH10B pTwist *tlpDΔN5*	KO1928	*rdxA::tlpDΔN5 3xflag erm*	This study
DH10B pTwist *tlpDΔC3*	KO1929	*rdxA::tlpDΔC3 3xflag erm*	This study
DH10B pTwist *tlpDΔC4*	KO1930	*rdxA::tlpDΔC4 3xflag erm*	This study
DH10B pTwist *tlpD*	KO1936	*rdxA::tlpD erm*	This study
pUT18	KO1346	BACTH vector	(30); J. Gober, UCLA
pUT18C	KO1347	BACTH vector	(30); J. Gober, UCLA
pKT25-zip	KO1348	BACTH pos. control vector	(30); J. Gober, UCLA
pUT18C-zip	KO1349	BACTH neg. control vector	(30); J. Gober, UCLA
XL1-Blue pUT18C-*tlpD*	KO1954	BACTH G27 *tlpD* cloned on the C-terminus of pUT18C	This study
XL1-Blue pUT18C-*tlpDΔC4*	KO1956	BACTH G27 truncated *tlpDΔC4* cloned on the C-terminus of pUT18C	This study
DH5a *cheV1*-pKNT25	KO1432	BACTH G27 *cheV1* cloned on the N-terminus of pKNT25	(16)
DH5a *cheW*-pKTN25	KO1434	BACTH G27 *cheW* cloned on the N-terminus of pKNT25	(16)

**TABLE 2:** Bacterial strains used in this study

Strain	KO reference no.	Genotype/description	Reference/source
G27	KO379		(31), Nina Salama
G27 *ΔtlpD*	KO1165	*rdxA::kan-sac ΔtlpD::cat*	(32)
G27 *ΔtlpD, rdxA::tlpD 3xflag*	KO1931	KO1165 transformed with pTwist *tlpD 3xflag*	This study
G27 *ΔtlpD, rdxA::tlpDΔN4*	KO1932	KO1165 transformed with pTwist *tlpDΔN4*	This study
G27 *ΔtlpD, rdxA::tlpDΔN5*	KO1933	KO1165 transformed with pTwist *tlpDΔN5*	This study
G27 *ΔtlpD, rdxA::tlpDΔC3*	KO1934	KO1165 transformed with pTwist *tlpDΔC3*	This study
G27 *ΔtlpD, rdxA::tlpDΔC4*	KO1935	KO1165 transformed with pTwist *tlpDΔC4*	This study
*m*G27	KO625	Mouse adapted G27	(33)
*mG27 ΔtlpD*	KO1006	KO625 *ΔtlpD::cat*	(34)
*m*G27 *ΔABCD*	KO1021	*ΔtlpA ΔtlpB ΔtlpC::aphA3 ΔtlpD::cat*	(17, 35)
*m*G27 *ΔABCD, rdxA::tlpD*	KO1940	KO1021 transformed with pTwist *tlpD*	This study
*m*G27 *ΔABCD, rdxA::tlpDΔC4*	KO1941	KO1021 transformed with pTwist *tlpDΔC4 3xflag*	This study

### TlpD tolerates C-terminal but not the N-terminal truncations

**Fig 2:**
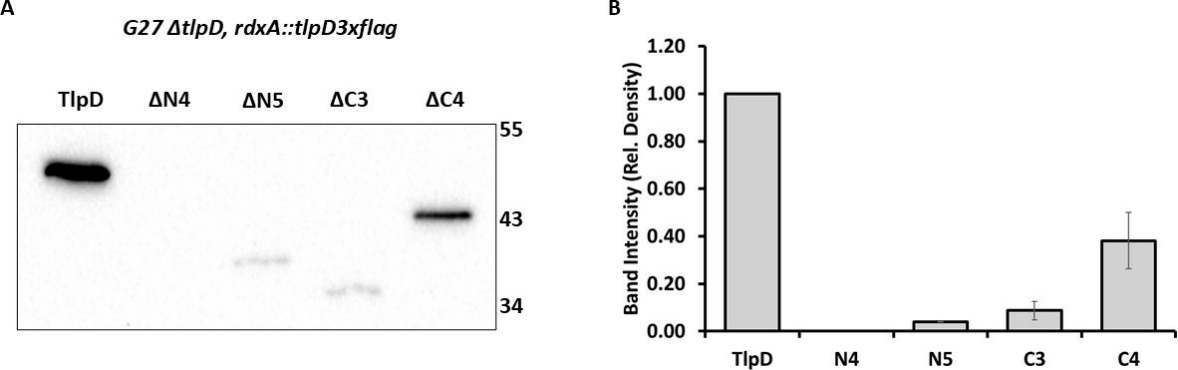
Analysis of abundance of TlpD WT and truncated variants encoded in *H. pylori rdxA*. (**A**) Western blot of *H. pylori* G27 ∆*tlpD* strains complemented with *tlpD* constructs was analyzed from whole-cell lysates with an anti-FLAG antibody, which recognizes the C-terminal TlpD tag. The TlpD variant is indicated at the top, with TlpD indicating full-length with the 3× FLAG. Marker sizes are given in kilodaltons on the right. The expected size of TlpD is 51 kDa, ΔN4 is 45 kDa, ΔN5 is 39 kDa, ΔC3 is 38 kDa, and ΔC4 is 45 kDa. Western blot is representative of three biological replicates. (**B**) Relative protein intensity from western blots. The TlpD variant quantified is indicated below, and the relative density is relative to full-length TlpD. The relative density is calculated from three biological replicates with error bars indicating the standard deviation.

To evaluate the production of these TlpD truncation constructs in *H. pylori*, each *tlpD* construct was transformed into *H. pylori* G27 *∆tlpD* (Table 2)*,* where they integrated into the genome at the *rdxA* locus (Fig. S2A). Proper integration was confirmed by PCR using primers that flank the *rdxA* locus (Fig. S2B). The presence of these proteins was evaluated in *H. pylori* whole-cell lysates by western blotting using an anti-FLAG antibody (Fig. 2). For this approach, *H. pylori* strains were grown on standard solid media (CHBA), scraped from the plate, normalized to a standard OD_600_, and lysed in Laemmli buffer. Full-length TlpD (TlpD) was detected when produced from the *rdxA* locus (Fig. 2), but strains producing the truncated variants showed variable production. N-terminus truncated proteins (TlpDΔN4 and TlpDΔN5) had little or no protein detected (Fig. 2). Strains encoding C-terminus truncated proteins (TlpD∆C3 and TlpD∆C4) varied in the amounts of protein detected: TlpDΔC3 had a low signal, similar to TlpDΔN5, while TlpDΔC4 had a clearly detectable signal that was approximately 38% of WT TlpD (Fig. 2). These results highlight the challenge associated with creating truncated proteins, but given that TlpD∆C4 was present in detectable amounts, this protein was chosen for further characterization to define functions for the C-terminal region.

### C-terminus truncated TlpDΔC4 retains interactions with CheV1 and CheW

**Fig 3:**
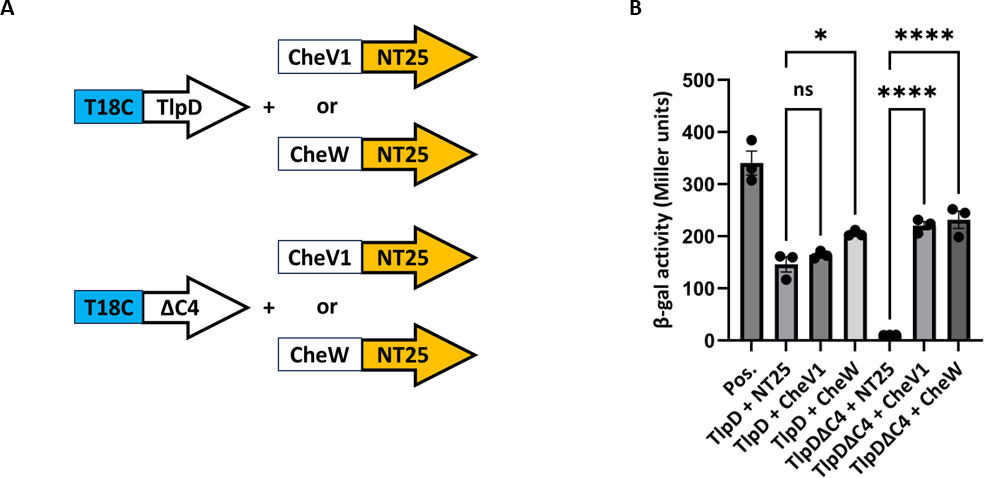
TlpD’s interaction with chemotaxis proteins is retained when the C-terminus is truncated. BACTH β-galactosidase (β-gal) assays of full-length and truncated (TlpDΔC4) TlpD with CheV1 and CheW. (**A**) Diagrams of constructs used. (**B**) β-gal assays. Positive (+) controls are T25-zip and T18-zip. Negative (−) controls are the respective TlpD construct with blank T25 plasmids. β-gal activity is calculated from three technical replicates with error bars indicating the standard error of the mean. *P < 0.05 and ****P < 0.0001.

We first evaluated whether there was evidence that TlpD∆C4 was able to fold by examining whether it retained aspects of normal function. TlpD is known to interact with chemotaxis proteins including the CheV and CheW coupling proteins (17). We therefore analyzed whether these interactions were retained for TlpD∆C4 using the bacterial two-hybrid (BACTH) approach previously used with *H. pylori* TlpD, CheV1, and CheW (16). Truncated *tlpD* was fused to the T18C fragment at the C-termini (T18C-*tlpDΔC4*), as done previously for full-length *tlpD* (T18C-*tlpD*), and then this construct was analyzed with previously used *cheV1* or *cheW* fused to the NT25 fragment (*cheV1*-NT25 or *cheW*-NT25) (Fig. 3A) (16). Positive control plasmids resulted in high β-galactosidase levels, while negative control plasmids without inserts displayed low levels, as expected (Fig. 3B). Full-length TlpD combined with CheW or CheV1 led to elevation of β-galactosidase activity significantly above that created with the empty plasmids, as seen previously (16). The T18-TlpD plasmid with the NT25 vector led to relatively high β-galactosidase activity that may be attributed to auto-activation. TlpDΔC4 was able to interact with both CheV1 and CheW as evidenced by β-galactosidase levels that were significantly above those of the two empty vectors or when TlpD∆C4 was present with empty pNT25 (Fig. 3B). Interestingly, the TlpD∆C4 construct had much lower auto-activation-type-β-galactosidase activity than full-length TlpD (Fig. 3B). Of note, these BACTH β-galactosidase levels are similar to those reported previously with full-length TlpD with CheV1 (16). Overall, this analysis supports that the truncated TlpDΔC4 retains the ability to bind both CheV1 and CheW, suggesting the TlpD∆C4 MA domain is likely correctly folded.

### TlpD∆C4 is unable to localize to the *H. pylori* cell pole

**Fig 4:**
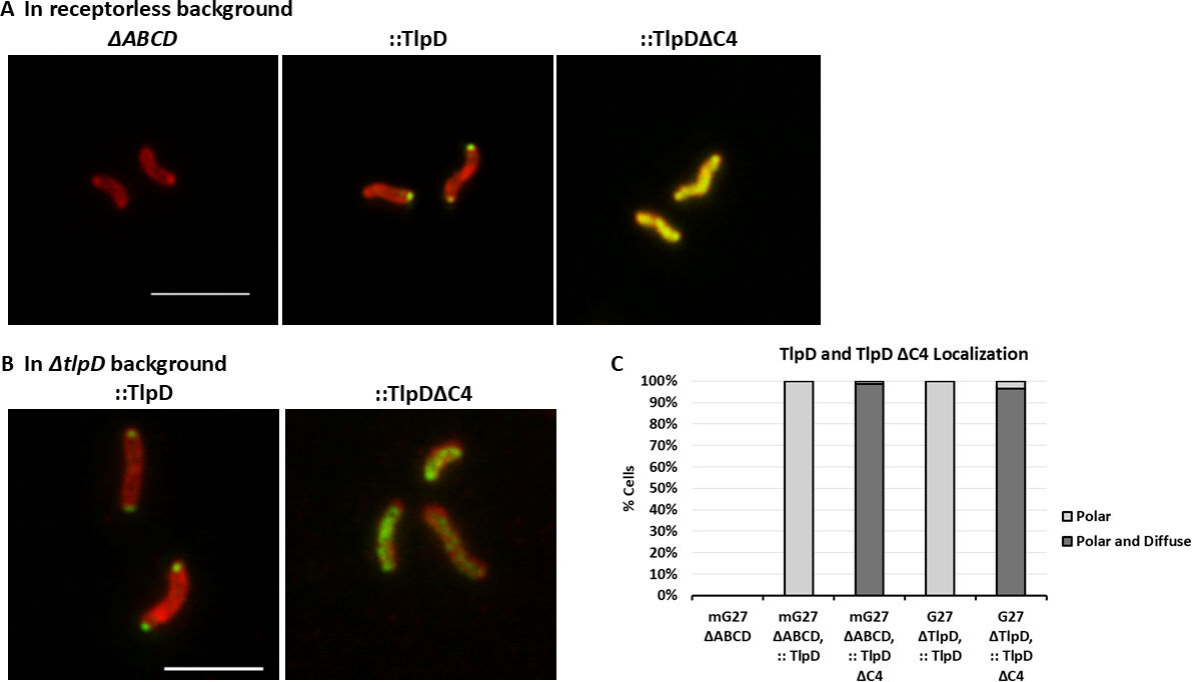
TlpD is polar when the C-terminus is intact and diffused when the C-terminus is deleted. Immunolocalization of TlpD or TlpD∆C4 in *H. pylori* whole cells. (**A, B**) *H. pylori* was detected with anti-*H*. *pylori* (red). TlpD proteins indicated above each image were detected with antibodies that detect all chemoreceptors (anti-TlpA22 or anti-FLAG (green). Scale bar = 4 μM. Images are representative of three biological replicates, with 250–300 bacterial cells analyzed for each sample. (**C**) Quantification of TlpD immunolocalization from the 250–300 cells are displayed as a percentage of cells with either only polar and no cytoplasmic staining (polar), or both polar and cytoplasmic staining (polar and diffuse).

TlpD normally localizes to the *H. pylori* pole even when it is the only chemoreceptor present (17). We next analyzed the localization of TlpD∆C4 using immunofluorescence when it was produced in either the presence of other chemoreceptors (TlpA and TlpB) or as the sole chemoreceptor. Full-length TlpD localized to the pole in the presence or absence of the other chemoreceptors, as previously shown for a strain of *H. pylori* with TlpD encoded at the native locus (17) (Fig. 4). Additionally, this result shows that an added FLAG tag does not disrupt TlpD localization, as the FLAG-tagged copy of WT TlpD used here localized as non-tagged TlpD, as previously reported (17). In contrast, TlpDΔC4 was diffuse throughout the cytoplasm (Fig. 4). This cytoplasmic localization did not change when the other chemoreceptors were present, although there might be some differences in the amount of TlpD in these two strain backgrounds (Fig. 4). These results thus suggest that TlpD∆C4 is unable to localize to the pole as full-length TlpD does.

### TlpD∆C4 alters the localization of chemotaxis coupling proteins CheV2 and CheV3

**Fig 5:**
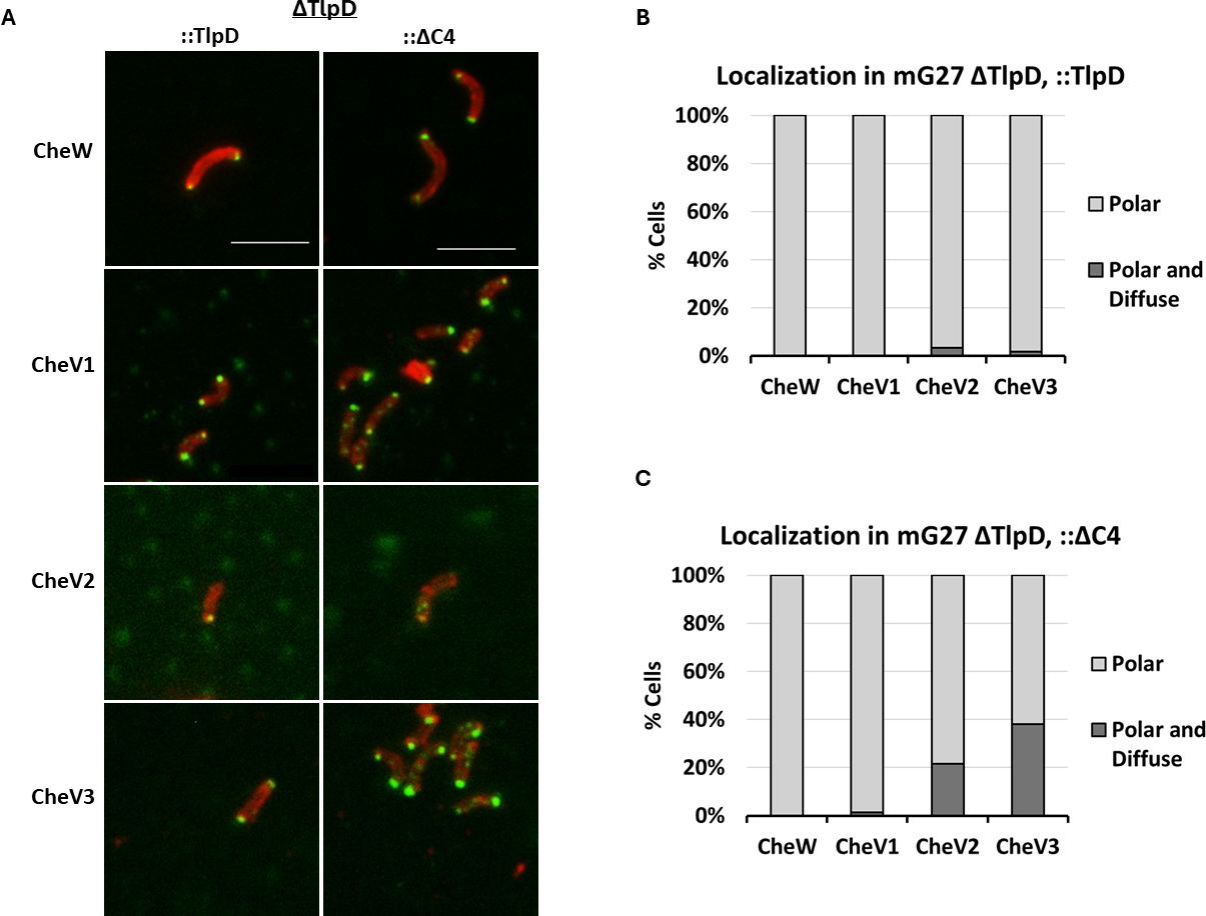
Localization of CheW and CheV coupling proteins in *H. pylori* in the presence of TlpDΔC4. (**A**) Immunolocalization of *H. pylori* chemotaxis proteins CheW, CheV1, CheV2, and CheV3 in whole cells producing full-length (TlpD-FLAG) or truncated (TlpDΔC4-FLAG) protein. *H. pylori* was detected with chicken anti-*H*. *pylori* (red). Individual chemotaxis proteins indicated on the left were detected with preabsorbed guinea pig anti-CheW, rabbit anti-CheV1, rabbit anti-CheV2, or rabbit anti-CheV3 (green). Scale bar = 4 μM. Images are representative of three biological replicates, with 250–300 bacterial cells analyzed for each sample. (**B**, **C**) Quantification of Immunolocalization results are displayed as a percentage of polar or polar and diffuse cells as in Fig. 4.

The above results provide support that TlpD∆C4 retained the ability to interact with chemotaxis coupling proteins (Fig. 3) but mislocalized (Fig. 4). CheV and CheW are normally localized to the cell pole in *H. pylori* by interactions with chemoreceptors and CheA (16, 17). We therefore analyzed whether TlpD∆C4 would affect the localization of these *H. pylori* chemotaxis signaling proteins, using immunofluorescence with antibodies specific to each coupling protein (17, 35). Full-length TlpD production resulted in CheV and CheW proteins being entirely or nearly entirely polar, as previously shown with WT *H. pylori* (17) (Fig. 5A and B). In contrast, CheV2 and CheV3 displayed elevated numbers of cells with both polar and nonpolar staining when TlpD∆C4 was produced, even in the presence of TlpA and TlpB (Fig. 5A). CheV3 showed the highest number of cells with diffuse cytoplasmic signal, rising from <5% to almost 40% (Fig. 5A and C). CheW and CheV1, in comparison, showed only polar staining (Fig. 5C). These results further support that TlpD∆C4 retains the ability to interact with chemotaxis coupling proteins and, furthermore, can compete with the polar TlpA and TlpB receptors to place CheV2 and CheV3 proteins in the cytoplasm.

### TlpDΔC4 does not confer soft agar migration in *H. pylori*

**Fig 6:**
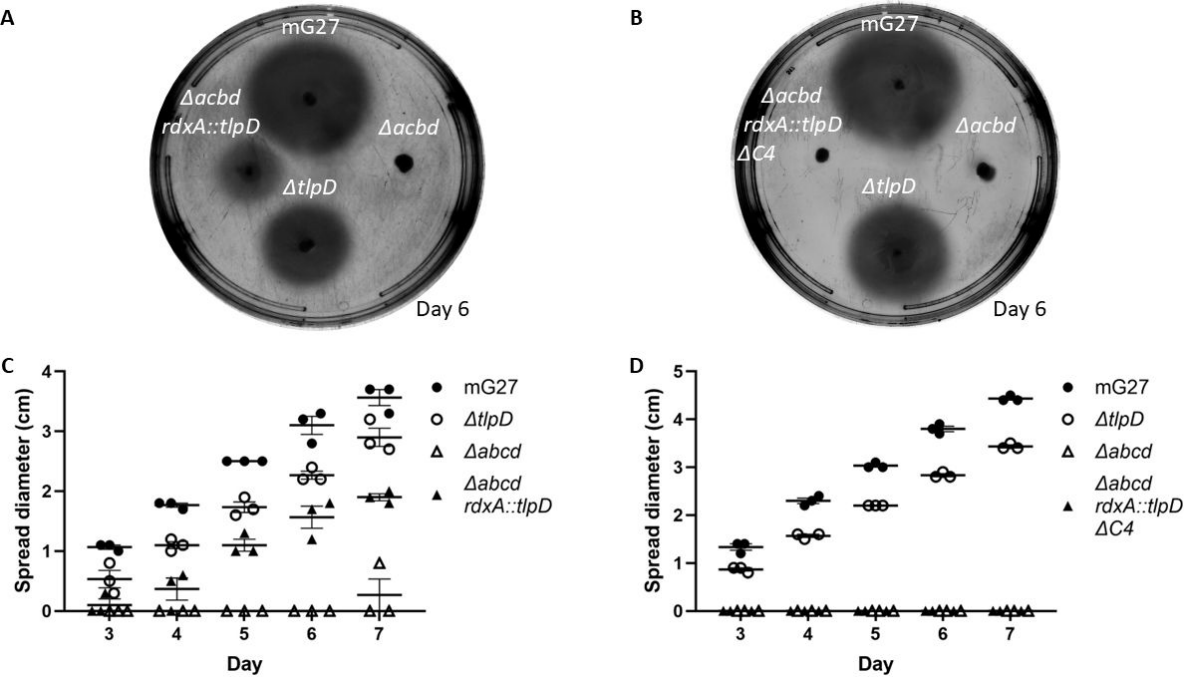
*H. pylori* that produce TlpDΔC4 as the sole chemoreceptor do not spread on soft agar. (**A**) *H. pylori* wild-type mG27 and isogenic mutants were analyzed for migration in soft agar. Strains were stabbed onto soft agar, and spreading was measured after 3 days. Strains included are the parent *H. pylori* lacking all chemoreceptors (*∆abcd*), and isogenic versions complemented with a full-length TlpD (*rdxA::tlpD*) (**A**) or with TlpDΔC4 (*rdxA::tlpD ΔC4 3xflag*) (**B**). Plates are representative of three biological replicates, with three technical triplicates. Error bars represent the standard error of the mean.

The above results showed that TlpD∆C4 was unable to locate to the pole. To explore TlpD∆C4 function, the chemotaxis ability of strains producing TlpD∆C4 were analyzed using a field-standard chemotaxis assay, migration in soft agar. In this assay, bacteria are inoculated at a single point, and if motile and chemotactic, migrate outwards to form expanded colonies. Using strains that produce the *rdxA* integrated copy of *tlpD* as their only chemoreceptor, we found that full-length TlpD was able to confer spreading to diameters that were significantly elevated compared to the parent strain lacking all chemoreceptors (*ΔABCD*) (Fig. 6A and C). These findings suggest that complementation of WT *tlpD* in the *rdxA* locus is able to restore spreading motility in *H. pylori,* although not quite to WT levels. The *tlpD*ΔC4 complemented strain, in contrast, did not spread even after seven days (Fig. 6B and D). This finding suggests that truncation of the C-terminus of TlpD results in a chemoreceptor that is insufficient to restore spreading, possibly because TlpDΔC4 may be unable to establish a working chemotaxis system.

## DISCUSSION

In this work, we created truncated versions of the *H. pylori* cytoplasmic chemoreceptor TlpD to analyze the functions of the N- and C-terminal regions. The functions of these TlpD regions were unknown. Our data provide support for the idea that TlpD’s C-terminal 45 amino acids play a role in polar localization, based on comparison of immunofluorescence of full-length TlpD versus TlpD∆C4. The full-length TlpD designed here, using a FLAG tag and integrated at a non-native site, was no different than the localization of natively expressed TlpD lacking the FLAG (17), suggesting the FLAG tag did not disrupt any localization signals. This result is consistent with those found with another C-terminal TlpD tag, V5 (36), suggesting adding sequence to the C-terminal end does not disrupt localization or function. TlpD∆C4, in contrast, did not display polar localization, suggesting that the C-terminal 45 amino acids are critical for localization directly or for allowing TlpD to adopt a form that can localize. Our analysis furthermore suggests that the TlpD∆C4 protein can fold, based on its ability to maintain coupling protein interactions in both *H. pylori* and the BACTH assays. Interestingly, two CheV proteins mislocalized to the cytoplasm more than other coupling proteins when TlpDΔC4 was present, suggesting that TlpD might have an interaction preference for these CheV proteins, as suggested by previous BACTH studies of TlpD (16) and seen in *Campylobacter jejuni* chemoreceptors (37). Overall, these findings support the new idea that the TlpD C-terminal 45 amino acids are required for localization.

One challenge of this work, and indeed any utilizing protein truncations, is that it’s difficult to remove portions of the proteins and preserve function. We made multiple attempts to remove portions of the TlpD N- or C-terminus, carefully identifying predicted domain boundaries, but most of our creations resulted in either no or very low protein production in *H. pylori*. Whether this outcome is due to poor translation, loss of protein stability, or some other reason is unknown. One truncation stood out, however, for its relatively robust levels of protein: ∆C4, the loss of the C-terminal 45 amino acids. It is not known why this particular construction resulted in more protein than the other mutants but less protein than WT; possibly, it is less stable or degraded due to partial misfolding. However, the ability to maintain interactions with known interacting proteins in two assays—BACTH and immunofluorescence—gave us confidence that we could learn of roles for the C-terminal region.

Although our data suggest the C-terminal domain is important for localization, no previously characterized domains or features were identified within it. The AlphaFold structure described here suggests that this region adopts an extended alpha helix that sits alongside the MA and CZB domains, with one side in a position to carry out protein-protein interactions and the other exposed on the surface (Fig. 1A). Based on the work here, a potential function of this C-terminal stretch is in promoting protein-protein interactions that in turn lead to localization. Other chemoreceptors have been shown to use the C-terminal region for promoting protein-protein interactions. Specifically, five amino acids at the C-terminal end of *E. coli* chemoreceptors Tar and Tsr promote protein-protein interactions with CheR and CheB (38–40). Thus, there is precedence for the chemoreceptor C-terminal region to play critical roles in functional interactions, possibly only using a relatively small set of amino acids.

Previous work suggested that TlpD’s polar localization was dynamic, finding that TlpD resided at the pole under conditions that correlated with high energy and was diffuse in conditions correlated with low ATP (25, 36). Besides the chemotaxis signaling proteins, several other proteins have been identified to interact with TlpD. Yeast two-hybrid analyses identified TlpD interactions with acetone carboxylase (AcxC, HP0599) (41) and sialic acid binding protein (HP0721) (42, 43). Two additional proteins, aconitase (AcnB) and catalase (KatA), were shown to interact with TlpD based on pull-downs from *H. pylori* extracts (36). AcnB, KatA, and AcxC were further studied for their ability to localize TlpD to the pole by analyzing TlpD’s position in mutants lacking the genes for each protein. The loss of *katA* resulted in TlpD being less polar and observed throughout the cell body (36). Whether TlpD interacts directly with KatA and via which regions is not known. This work, however, corroborates that TlpD is polarly localized in a manner that appears to be regulated and via specific protein-protein interactions beyond the chemotaxis signaling network.

TlpD is well known to play a critical role in building the *H. pylori* chemotaxis protein arrays. Previous work had shown that TlpD, even as the sole chemoreceptor, localizes to the pole and is sufficient to localize the main components of the chemotaxis signaling complex—CheA, CheW, CheV1, and CheV3 to the cell pole (17). Furthermore, work with *H. pylori* that produced only one transmembrane chemoreceptor, either TlpA or TlpB, and lacked TlpD mislocalized all three CheV proteins (44). These studies suggest TlpD plays a critical role in promoting chemotaxis protein localization to the pole. Our localization studies showed that even when using an *H. pylori* strain that retained TlpA and TlpB, two transmembrane chemoreceptors, the TlpD∆C4 was not at the pole and mislocalized CheV proteins (Fig. 5). These findings suggest that transmembrane chemoreceptors of *H. pylori* are not sufficient to maintain a polar chemotaxis complex but rather require polar TlpD.

These findings may provide insight into the workings of the many cytoplasmic chemoreceptor proteins (2). Multiple cytoplasmic chemoreceptors localize to the cell pole and have been shown to do so by colocalization with transmembrane chemoreceptors (45–47), and in some cases, switch between a diffused cytoplasmic protein and a distinct polar topology (36, 48). Cytoplasmic chemoreceptors presumably utilize similar mechanisms as those used by other proteins to drive polar localization, including stochastic self-assembly, interaction with polar anchor proteins, or interactions with plasmid partitioning homologs (49). There are numerous other CZB domain-containing chemoreceptors, so it’s possible our findings may be applicable to these (2). Most of these remain unstudied, however. One candidate for comparative analysis is the CZB-containing cytoplasmic chemoreceptor McpA (NCBI Ref Seq: WP_000475246.1) from the enteric pathogen *Salmonella enterica*. McpA is a 352 amino acid chemoreceptor protein with a central MA domain, a C-terminal CZB domain (residues 246-303), and a C-terminal coiled coil that extends beyond the MA domain, of as-yet-undefined function (50, 51). The alignment of full-length TlpD and McpA shows 33% amino acid identity over the entire protein, and the C-terminal domains, which include the CZB domain, share 29% amino acid identity, but there is no specific similarity at the extreme C-terminus. The localization of McpA is not yet known, however, and will be interesting for future studies.

In sum, our work shows that TlpD supports the building of a functional chemosensory complex at the pole and relies on the sequences at its C-terminal end to accomplish this function. One model, consistent with our data, is that the C-terminus of TlpD interacts with an as-yet-identified polar landmark protein to bring TlpD to the pole, possibly via one of its previously identified binding partners such as catalase. Once there, TlpD plays a critical role in nucleating chemosensory array formation. Testing this model and exploring whether other cytoplasmic chemoreceptors have similar localization sequences is an exciting area for future work.

## MATERIALS AND METHODS

### Bacterial strains and growth conditions

All *H. pylori* and *E. coli* strains used in this study are listed in Tables 1 and 2*. H. pylori* was grown on Columbia Horse Blood Agar (CHBA) containing 5% (vol/vol) defibrinated horse blood (Hemostat Labs, Davis, CA, USA), 0.2% (wt/vol) β-cyclodextrin (TCI), 5 μg/mL cefsulodin (Sigma Aldrich), 50 μg/mL cycloheximide (GoldBio), 2.5 U/mL polymyxin B (Sigma Aldrich), and 10 μg/mL vancomycin (GoldBio). For the selection of *H. pylori* mutants, 13 μg/mL chloramphenicol, 15 μg/mL kanamycin (Fisher BioReagents), or 25 μg/mL erythromycin (GoldBio) was used. For liquid cultures, *H. pylori* was grown with Brucella broth containing 10% heat-inactivated fetal bovine serum (Gibco) (BB10). Cultures were incubated at 37°C in microaerobic conditions (5% O_2_, 10% CO_2_, and 85% N_2_). *E. coli* was grown on LB media containing 100 μg/mL ampicillin or 30 μg/mL kanamycin (Fisher BioReagents) and incubated at 37°C unless otherwise stated.

### Strategy for creation of truncated TlpD

To construct truncated versions of G27 TlpD (Uniprot: B5Z6X0), ProteinCCD (52) was used to identify predicted secondary structures, features, and domains within the protein and served as the basis for selecting truncation sites that avoid secondary structures and predicted or known regions. TlpD N-terminal truncations at amino acids 53 (ΔN4) and 104 (ΔN5) were selected to sit between secondary structures. The TlpD C-terminus truncations at amino acids 322 (ΔC3) and 388 (ΔC4) were selected to abolish or retain the CZB domain without interrupting the canonical MA domain, respectively. Additionally, 200 bp upstream of *tlpD* was included to capture any transcriptional regulatory sites, and sequences encoding a 3× FLAG tag (DYKDDDDK-DYKDDDDK-DYKDDDDK) were added to the C-terminus of TlpD. Finally, an erythromycin resistance gene and its promoter were added for positive selection, downstream of *tlpD*. This entire sequence was flanked by 500 bp upstream and downstream of the non-native locus, *rdxA*, for chromosomal integration in *H. pylori*. pTwist Amp High Copy plasmids with ampicillin resistance were ordered from Twist Bioscience, which contained these *tlpD* cassette sequences (File S2). These plasmids were then transformed into electrocompetent *E. coli* DH10B cells using ampicillin selection followed by single-colony purification.

### Creation of G27 and mG27 *tlpD* complement mutants

For transformation into *H. pylori*, plasmids containing *tlpD* sequences were purified from *E. coli* DH10B cells using the QIAprep Spin Miniprep Kit (Qiagen) and added to a suspension of BB10 and 1-day-old *H. pylori* G27 *ΔtlpD* or *ΔtlpA ΔtlpB ΔtlpC ΔtlpD* cells from a CHBA plate (53). Transformants were selected by growth on CHBA containing 25 μg/mL erythromycin, followed by single-colony purification. All mutations were verified using PCR with primers that flank *rdxA* (*rdxA* Fwd and Rev) (Table S1).

### Western blotting

For proteins from *E. coli*, liquid cultures were grown overnight and used to prepare lysate samples. For *H. pylori*, 1- to 2-day-old samples grown on CHBA solid media containing erythromycin were resuspended in 1× phosphate-buffered saline (PBS) and used to prepare lysate samples. To prepare the lysate, samples were back-diluted to OD_600_ = 0.7 in 200 μL. 6× SDS PAGE sample buffer was added to the mixture at 1× final concentration, and 1× PBS was added to 200 μL. Samples were heated at 95-100°C for 10 min and placed on ice. Samples were electrophoresed on 10% or 12% SDS-PAGE gels and semi-dry transferred to polyvinylidene (PVDF) membranes using a trans-blot SD semi-dry transfer cell (Bio-Rad). Relative protein loading was visualized by staining the membrane with Direct Blue 71 (DB71) stain containing 0.008% DB71 (Sigma Aldrich) in 40% EtOH and 10% acetic acid prior to antibody binding. PVDF membranes were incubated with a 1:20,000 dilution of rat anti-FLAG monoclonal antibody (Novus Biological, NBP1-06712) or a 1:4,000 to 1:10,000 dilution of rabbit anti-TlpA22 antibody (32) diluted in 5% BSA at 4°C overnight. Membranes were next incubated with a 1:20,000 dilution of horseradish peroxidase-conjugated goat anti-rat (R&D Systems) or a 1:10,000 dilution of goat anti-rabbit (Santa Cruz Biotechnology) secondary antibody. For visualization, SuperSignal West Pico PLUS Chemiluminescent Substrate (Thermo Scientific) was added at a 1:1 ratio for 30–120 s with shaking at room temperature. Blots were then imaged using a Bio-Rad Chemi-doc. Quantification of bands was carried out using Fiji ImageJ software for normalization of peaks to the area of the full-length TlpD peak. The band intensity of the TlpD truncation constructs is relative to the full-length TlpD construct.

### Creation of bacterial two-hybrid (BACTH) plasmids and BACTH analysis

Synthetic plasmid DNA from *E. coli* DH10B was extracted using the Qiaprep Mini Kit (Qiagen). *H. pylori* G27 full-length *tlpD* and truncated *tlpDΔC4* constructs were amplified using PCR with primers that amplify each sequence (*tlpD* pUT18 Fwd and Rev, *ΔC4* pUT18 Fwd and Rev) (Table S1). The PCR products encoded PstI and SacI restriction sites, which were digested with PstI-HF (NEB) and SacI-HF (NEB) restriction enzymes. BACTH plasmids pUT18 and pUT18C (J. Gober, UCLA) (30) were similarly digested, followed by phosphatase treatment using rSAP following the manufacturer’s suggested protocol (NEB). Treated PCR products and plasmids were purified using the GFX PCR DNA and Gel Band Purification Kit (Cytiva). The purified products were ligated using T4 DNA ligase following the manufacturer’s suggested protocol (NEB) and subcloned into electrocompetent *E. coli* XL1-Blue cells, and ampicillin-resistant colonies were selected. All constructs were confirmed using PCR with the same primers used to amplify *tlpD* or *tlpDΔC4* (Table S1) and DNA sequencing (Azenta).

Recombinant plasmids encoding the *cheV1*-NT25 (16), *cheW*-T25 (16), T18C-*tlpD*, and T18C-*tlpDΔC4* were co-transformed, at 40 ng each, into electrocompetent BTH101 and plated on LB containing 40 μg/mL X-gal (5-bromo-4-chloro-3-ondolyl-beta-galacto-pyranoside) (GoldBio), 0.5 mM IPTG (Isopropyl β-d-1-thiogalactopyranoside) (Fisher BioReagents), 100 μg/mL ampicillin, and 50 μg/mL kanamycin. As positive controls, T25-zip and T18-zip plasmids were co-transformed into BTH101. As negative controls, each *tlpD* recombinant plasmid was co-transformed with a pKNT25 blank BACTH plasmid. For interaction assays, colonies, including controls grown on LB containing X-gal, IPTG, ampicillin, and kanamycin at the above-mentioned concentrations, were used to make overnights in LB with ampicillin and kanamycin at the above-mentioned concentrations. The following day, 20 μL of each overnight culture was dropped onto LB plates containing X-gal, IPTG, ampicillin, and kanamycin as above. These spots were allowed to dry at room temperature before the plates were incubated at 30°C for 24 h. Images were captured using an iPhone 14 Pro and a white light transilluminator (Fisher Scientific). Images are representative of three biological replicates.

### β-Galactosidase assay

To quantify BACTH interactions, β-galactosidase activity was measured as previously described (16, 30). Briefly, transformants were grown in LB broth with ampicillin, kanamycin, and IPTG at the above concentrations at 30°C overnight. Overnight cultures were back-diluted to OD_600_ = 0.1 in LB broth with ampicillin, kanamycin, and IPTG, then grown at 30°C until OD_600_ 0.3–0.7 was reached. 100 μL of each culture was mixed with 900 μL cold Z-Buffer (0.06 M Na_2_HPO_4_·7 H_2_O, 0.04 M NaH_2_PO_4_–H_2_O, 0.01 M KCl, 0.001 M MgSO_4_·7H_2_O, adjusted to pH 7.0, and 0.05 M β-mercaptoethanol was added fresh before use) in triplicate and mixed by inversion. Next, 50 μL of 0.1% SDS and 100 μL of chloroform were added to each sample followed by a 10-s vortex. The samples and fresh 4mg/mL ortho-Nitrophenyl-β-galactoside (ONPG) (Thermo Scientific) in 0.1 M Phosphate Buffer solution (0.06 M Na_2_HPO_4_·7H_2_O and 0.04 M NaH_2_PO_4_·H_2_O adjusted to pH 7.0) were incubated at 30°C for 5 min. 200 μL ONPG solution was added to the samples, vortexed, and the time was recorded. The samples were incubated at 30°C until a yellow color appeared. The reactions were stopped by adding 500 μL Stop solution (1 M Na_2_CO_3_), time recorded, and samples centrifuged at room temperature and maximum speed for 5 minutes before OD_420_ was measured. Miller units were calculated to determine β-galactosidase activity using the following formula: 1,000 × (OD_420_/[time × vol. culture × OD_600_]) (54). β-gal activity is one biological replicate with three technical replicates per sample. Statistical analysis was done on GraphPad Prism v9.4.1.

### Immunofluorescence

Immunolocalization of *H. pylori* chemotaxis proteins TlpD, CheW, CheV1, CheV2, and CheV3 was performed as previously described (17, 35). Briefly, a 1-day-old *H. pylori* grown on CHBA under microaerobic conditions was harvested and resuspended in 1 mL of 1× PBS. 20 μL of the cell suspension was placed on a Poly-Lysine glass slide (Fisher Scientific) and fixed with 0.075 M NaPO_4_ adjusted to pH 7.4, 0.0025 M NaCl, and 2% paraformaldehyde (EMS) solution then permeabilized in 3% bovine serum albumin (BSA) (Millipore), 1% Saponin (Calbiochem), 0.01% Triton X-100 and 0.02% Na Azide (Sigma Aldrich) in 1× PBS. The samples were blocked with 3% BSA and 0.01% Triton X-100 in 1× PBS solution. Samples were next treated with a 1:500 dilution of chicken anti-*H*. *pylori* (Agrisera AB), a 1:2,000 dilution of rat anti-FLAG (Novus Biological, NBP1-06712), a 1:200 dilution of preabsorbed or non-preabsorbed rabbit anti-TlpA22, or a 1:50 dilution of preabsorbed rabbit anti-CheV1 (35), anti-CheV2, anti-CheV3 (J. Castellon, P. Lertsethtakarn, and K.M. Ottemann, unpublished), or guinea pig anti-CheW (17) diluted in permeabilization solution (described above). The anti-TlpA22, anti-CheV1, anti-CheV2, and anti-CheV3 antibodies were previously shown to be specific to each coupling protein (17, 35). The samples were then treated with a 1:500 dilution of goat anti-chicken Alexa Fluor 594 (Invitrogen, A11042) and goat anti-rat Alexa Fluor 488 (ThermoFisher, A48262) or a 1:300 dilution of goat anti-rabbit Alexa Fluor 488 (Invitrogen, A11008) or goat anti-guinea pig Alexa Fluor 488 (Invitrogen, A11073) diluted in permeabilization solution. The samples were washed with blocking solution (described above) three times, a drop of Vectashield (Vector Laboratories) was added to the cells, and then sealed with coverslips. Fluorescence microscopy, using the Nikon Eclipse E600, was used to visualize immunofluorescent cells at 100× objective with oil immersion. Texas Red and FITC/GFP filters were used to separately view and capture emission spectra from Alexa Fluor 594 (red) and 488 (green), respectively. Images were captured using a Hamamatsu ORCA-285 digital camera equipped to the microscope. Fluorescent images were pseudo-colored using ImageJ and merged using Adobe Photoshop version 24.1.0. Scale = 4 μm. Images are representative of three biological replicates. % cells were calculated to determine the number of polar or polar and diffuse cells by counting 250–300 cells across three biological replicates.

### Soft-agar motility assay

*H. pylori* spreading motility was carried out as previously described (17, 55–57). Briefly, soft agar plates were made with Brucella broth and 0.35% Bacto agar (BD). Soft agar was cooled to 50°C before 2.5% heat-inactivated fetal bovine serum, 8 μg/mL amphotericin B, 5 μg/mL cefsulodin, 50 μg/mL cycloheximide, 2.5 U/mL polymyxin B, 5 μg/mL trimethoprim (Sigma Aldrich), and 10 μg/mL vancomycin were added. Plates were allowed to cure for 3 days at room temperature before use. 1- to 2-day-old *H. pylori* strains from CHBA plates were inoculated halfway into the agar using a pipette tip. Plates were incubated right-side up in a 37°C microaerobic chamber, and migration diameter was measured daily. mG27, mG27 *ΔtlpD*, and mG27 *ΔtlpA ΔtlpB ΔtlpC ΔtlpD* were used as controls. Statistical analysis was done on GraphPad Prism v9.4.1.

## SUPPLEMENTAL MATERIAL

File S1 jb.00394-25-s0001.txt TlpD secondary structure bioinformatic analysis. Outputs from bioinformatics analysis.

File S2 jb.00394-25-s0002.txt tlpD plasmid sequences.DNA sequences of the tlpD-containing inserts and full constructs created for this work.

Supplemental figures and tables jb.00394-25-s0003.docx Figures S1 to S3 and Table S1.

ASM does not own the copyrights to Supplemental Material that may be linked to, or accessed through, an article. The authors have granted ASM a non-exclusive, world-wide license to publish the Supplemental Material files. Please contact the corresponding author directly for reuse.
