# Supplementary material for: The Helicobacter pylori TlpD cytoplasmic chemoreceptor requires an intact C-terminus for polar localization and function
Source: J Bacteriol. 2026 Feb 9;208(3):e00394-25. doi: 10.1128/jb.00394-25 (PMC13001251; doi:10.1128/jb.00394-25)
Supplement: Supplemental figures and tables — Figures S1 to S3 and Table S1. [file jb.00394-25-s0003.docx]

# Supporting information


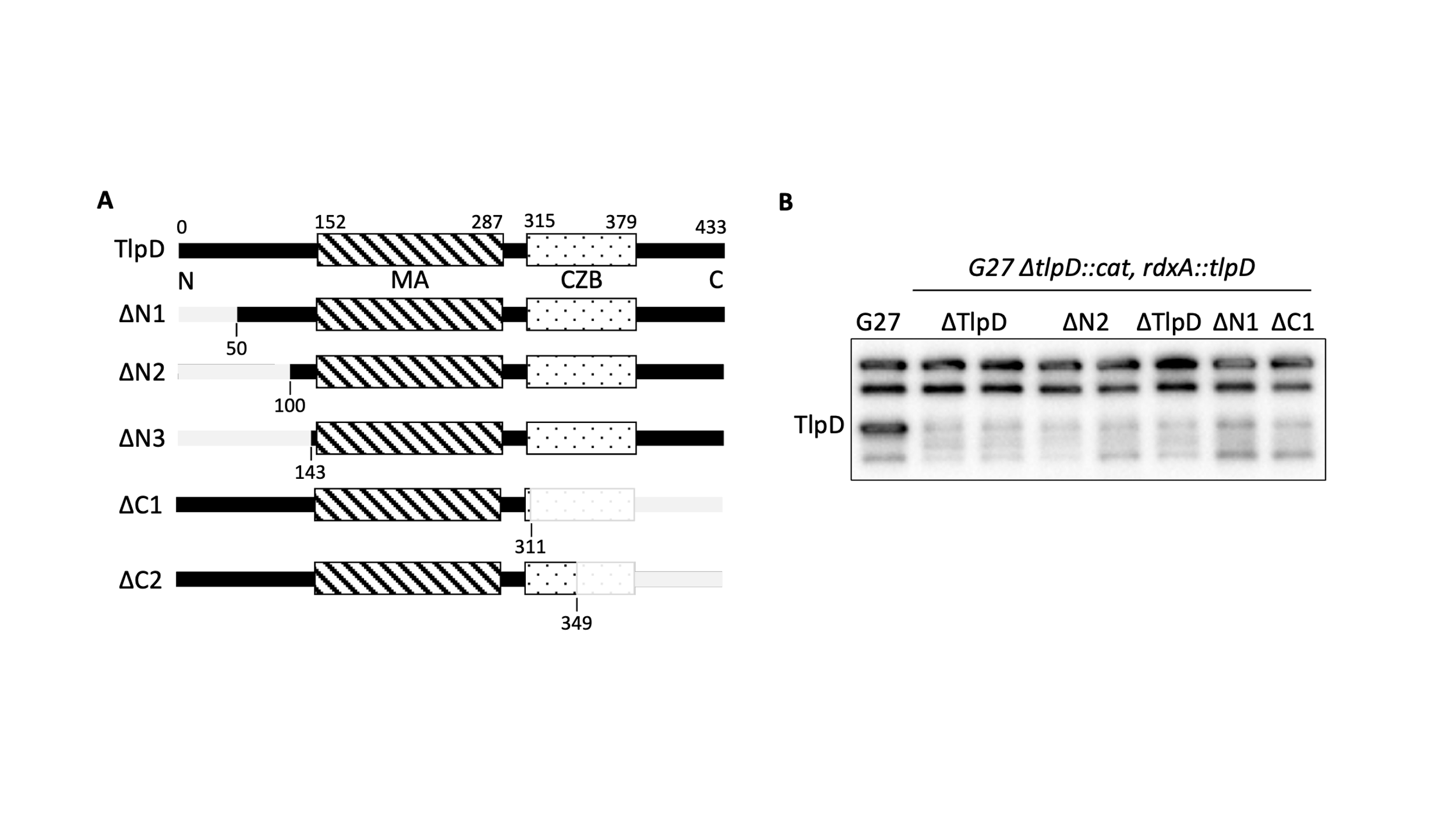


**Figure S1. First generation TlpD truncation constructs and production in H. pylori.** (A) Modified versions of *H. pylori* TlpD with domains and truncation sites (grey). The top line “TlpD” is the full-length version of the protein at 433 amino acids, ΔN1 is truncated at residue 50, ΔN2 is truncated at residue 100, ΔN3 is truncated at residue 143, ΔC1 is truncated at residue 311, and ΔC2 is truncated at residue 349. (B) Western blot of *H. pylori* G27 strains with complemented *tlpD* constructs were analyzed from whole-cell lysates with an anti-TlpA-22 antibody (33) which recognizes the conserved MA domain of all chemoreceptors. The expected size of wild-type TlpD is 48 kDa, ΔN1 is 42.9 kDa, ΔN2 is 37.4 kDa, and ΔC1 is 34 kDa. ΔTlpD lanes are the parent strain with no integrated *tlpD*. The two bands above TlpD are TlpA and TlpB. Western blots are representative of 3 biological replicates.


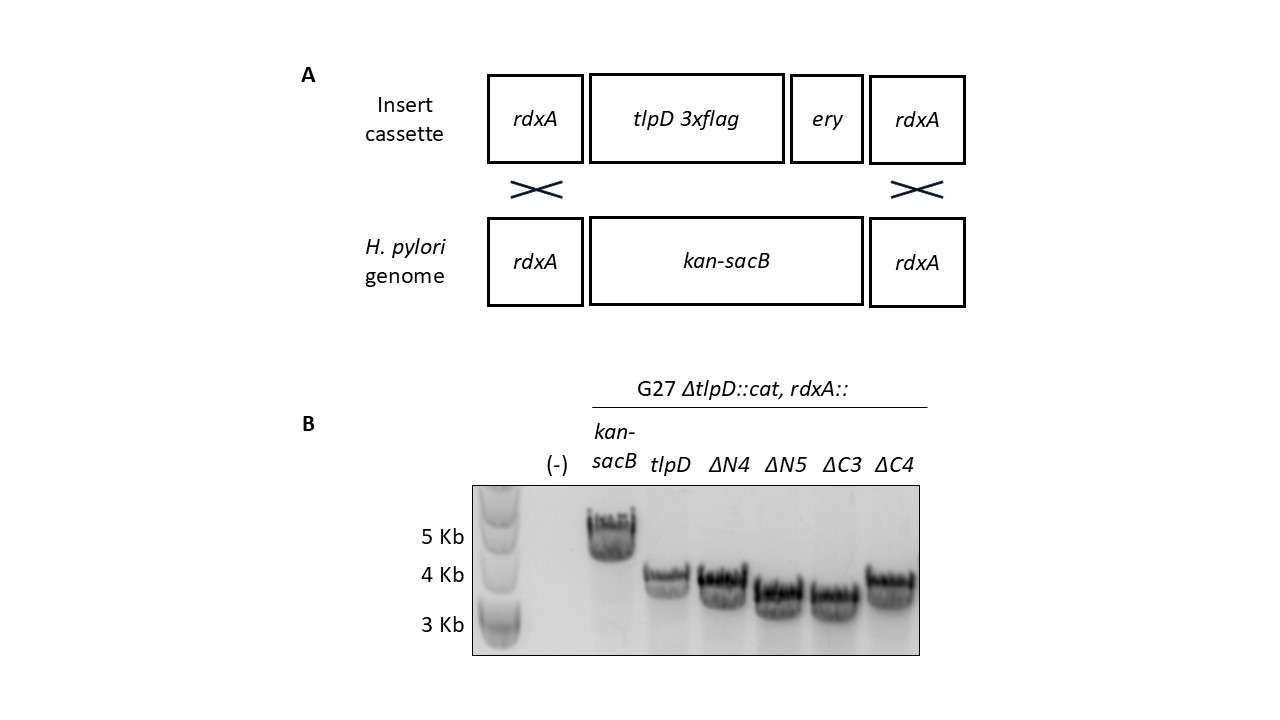


**Figure S2. tlpD sequence layout for placement into the H. pylori genome.** (A) Design of the *tlpD* insert cassette for integration into the *H. pylori* chromosome. This cassette contains the *tlpD* sequence, a sequence encoding a C-termina *3xflag* tag, and erythromycin (*ery*) resistance sequences that are flanked by *rdxA* homologous regions for integration into the *H. pylori* genome at the *rdxA* locus. (B) PCR amplification of erythromycin-resistant *H. pylori* isolates using primers that flank the *rdxA* locus. The nomenclature above each band indicates the amplified product. (-); PCR no template control. *kan-sacB*; kanamycin resistance and sucrose sensitivity selection marker present in the *H. pylori* transformation background that serves as a negative control. *tlpD*, ΔN4, ΔN5, ΔC3, and ΔC4 are the versions of *tlpD* on the cassette that were PCR amplified as shown in Fig. 1B.


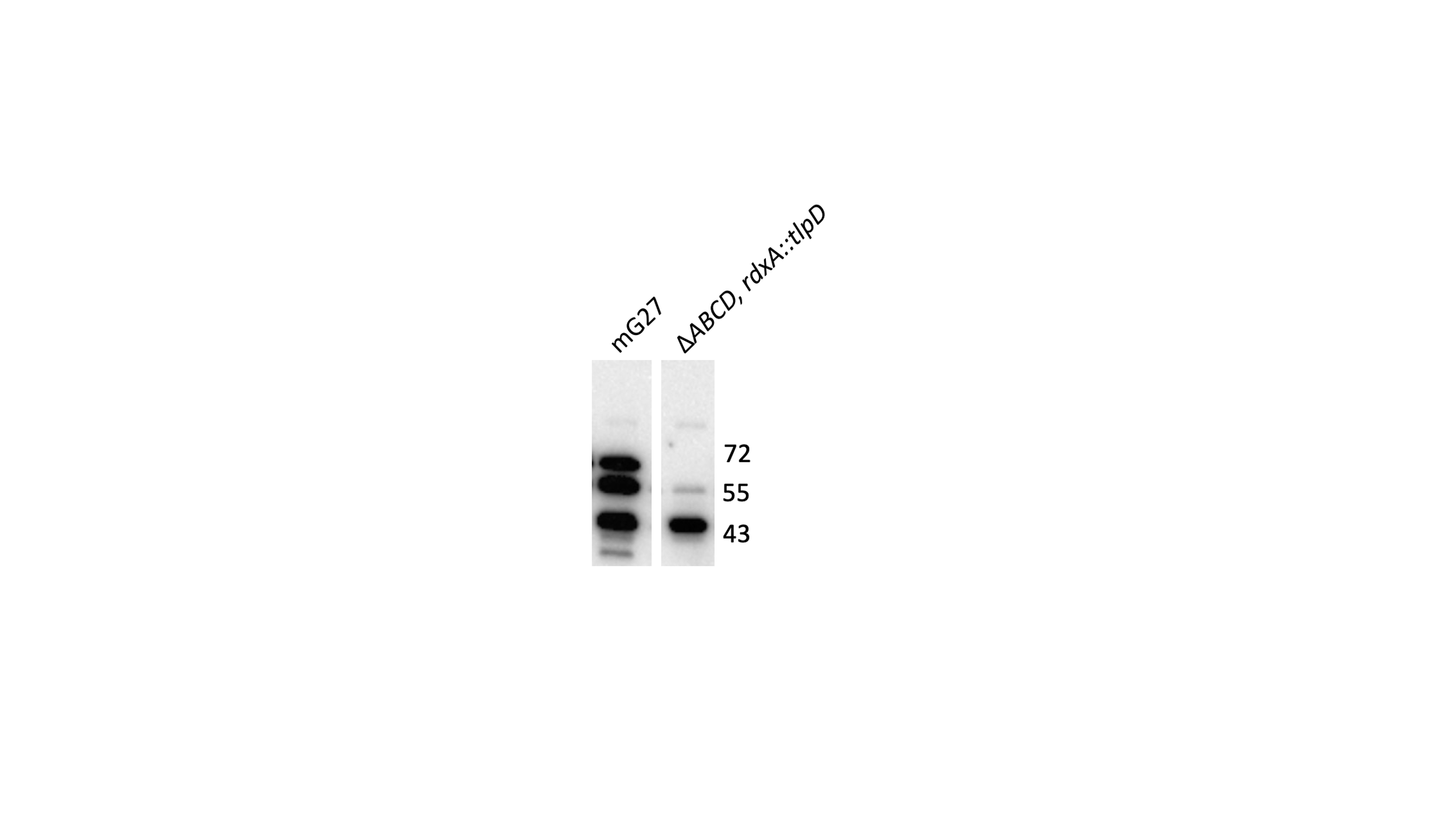


**Figure S3. TlpD is produced when complemented into *H. pylori* that lacks all chemoreceptors.** Western blot of *H. pylori* mG27 *Δabcd* producing complemented WT *tlpD* was analyzed from whole-cell lysates using an anti-TlpA22 antibody, which recognizes the MA domain. The strains analyzed are indicated at the top. Marker sizes are given in kilodaltons on the right. The expected size of TlpD is 48 kDa. Western blot is representative of 2 biological replicates.

| **Table S1. Primers used in this study.** | |
| --- | --- |
| **Primer** | **Oligonucleotide sequence** |
| rdxA Fwd | cgccattcttgcaagatgtttg |
| rdxA Rev | ctcgcttctgccaccctctt |
| *tlpD* pUT18 Fwd | agatCTGCAGatgtttgggaataagcagttac^a^ |
| *tlpD* pUT18 Rev | ggagGGTACCttcgcctttttgaattttttcaatg^a^ |
| *ΔC4* pUT18 Fwd | aattCTGCAGatgtttgggaataagcagttacagc^a^ |
| *ΔC4* pUT18 Rev | gagaGGTACCggtaatgtggtcttcttgaacg^a^ |

^a^Restriction sites are capitalized.

**File S1. TlpD secondary structure bioinformatic analysis.**

Outputs from bioinformatics analysis.

**File S2. *tlpD* plasmid sequences.**

DNA sequences of the *tlpD*-containing inserts and full constructs created for this work
